# Challenges experienced by grandparents caring for AIDS orphans in the Western Cape province, South Africa

**DOI:** 10.4102/hsag.v29i0.2454

**Published:** 2024-04-30

**Authors:** Furaha Akimanimpaye, Million S. Bimerew, Deliwe R. Petlhu

**Affiliations:** 1 School of Nursing, Faculty of Community Health Sciences, University of the Western Cape, Cape Town, South Africa; 2 Department of Nursing Science, Faculty of Health Care Sciences, Sefako Makgatho Health Sciences University, Ga-Rankuwa, Pretoria, South Africa

**Keywords:** HIV and AIDS, grandparents, orphans, support, social support, emotional support, stigma, well-being

## Abstract

**Background:**

There is limited understanding of the difficulties and experiences faced by grandparents who assume the responsibility of caring for AIDS orphans.

**Aim:**

The objective of the study was to investigate and depict the difficulties encountered by grandparents who provide care for AIDS orphans in the Western Cape province of South Africa.

**Setting:**

The study was conducted in the City of Cape Town Metropolitan and the Overberg Municipality.

**Methods:**

The study used qualitative approach with an exploratory-descriptive design. A purposive sampling technique was utilised to select 25 grandparents. Semi-structured interviews were conducted, audio-recorded, transcribed verbatim, and analysed thematically using ATLAS.ti, version 7.

**Results:**

Financial difficulties, a lack of support, HIV and/or AIDS stigma, and dealing with rebellious teenagers were primary challenges affecting the well-being of grandparents.

**Conclusion:**

The study found that while financial challenges were significant, other factors such as poor family support contributed to the decline in the well-being of grandparents caring for AIDS orphans.

**Contribution:**

The study’s results can lead to improved public health programmes that address the identified challenges and health needs of grandparents providing care to AIDS orphans and the orphans under their care.

## Introduction

The prevalence of AIDS remains a significant contributor to global mortality rates, with South Africa being home to approximately 8.2 million people living with HIV (Stats SA 2022). One of the grim consequences of the HIV epidemic is the death of young parents who leave behind minor children below the age of 18 years who rely on others for care and support. The snowball effect is the shift of responsibility to the older relatives, particularly grandparents, who are assumed to be the ideal primary guardians of these orphans (Osafo et al. 2017). However, the adverse impact of the AIDS epidemic on the health and wellness of these grandparents, who are tasked with assuming the full burden of caregiving, has been repeatedly observed over the years (Kidman & Thurman 2014). Caregiver burden and the financial strain burden on grandparents caring for AIDS orphans have a ripple effect on their overall well-being, affecting not only their finances but also their physical health, social connections, and emotional state (Matovu, Rankin & Wallhagen 2020; Zimmer & Das 2014).

Caring for orphans, especially the ones living with AIDS involves various undertakings because they often have special needs that intensify caregiving responsibilities (Böning & Ferreira 2013; Shaibu 2016). Given their advanced age, providing care to their grandchildren puts additional stress on the well-being of grandparents, who are already dealing with their own emotional, physical, and nutritional challenges, and limited resources, particularly finances. The daily responsibilities of caregiving affect their overall welfare as they encounter numerous difficulties, including physical and psychological challenges such as body aches and back pain, stress, anxiety, and depression. In addition, unfavourable living conditions such as inadequate housing and overcrowding, and socio-cultural barriers such as stigma, prejudice, and inadequate support add to their woes (Bartlett 2019; Kalomo & Liao 2018; Kalomo & Tauken 2022).

Grandparents who take care of AIDS orphans bear a significant weight that encompasses not only the stigma and discrimination they face but also the impact of policies that overlook their involvement in HIV and/or AIDS.

Limited access to support services is often perpetuated by discriminatory policies as they exclude grandparents from the benefits that are available to others such as the foster grant system (Saldulker & South Gauteng High Court 2012). Such policies do not take into consideration that caring for AIDS orphans is a continuous activity, which in essence would have fallen in the ambit of government. Hence, grandparents need significant and ongoing support. Various entities including governments, non-governmental organisations (NGOs), and community organisations need to enhance their caregiving abilities for AIDS orphans. To address and uphold the rights of orphans, the South African government implemented a social welfare system aimed at offering various social grants to provide financial support to households and foster families responsible for their care (Breckenridge et al. 2019). However, the eligibility requirements for foster grants, as outlined in the current policy (*The Children’s Act 38 of 2005*), impose restrictions on grandparents’ access to these grants because of their status as the natural parents responsible for the orphans. This means that when an orphan lives with a grandparent, they are only eligible for the Child Support Grant, which provides less financial support than the Foster Care Grant. Although the eligibility criteria can be relaxed if relevant documents such as birth certificates and identity documents are submitted and evidence of inability to support the grandchild is provided, this often poses a huge challenge for the grandparents whose income tithers on the eligibility cut-off amount (Hall, Skelton & Sibanda 2016). Furthermore, grandparents frequently encounter challenges while attempting to access this support, as the administrative procedures can be complicated and time-consuming. The procedure can become even more problematic when the grandparent is not able to provide the required documents, which may not be readily available during the application process (Breckenridge et al. 2019; Damian, Mashau & Tugli 2019; Kadungure 2017).

In South Africa, grandparents taking care of AIDS orphans can receive additional assistance, including counselling, food donations, and educational aid, through NGOs. However, such forms of support are unreliable, as their provision depends upon funding availability, thereby leaving grandparents with a significant burden to bear.

### Problem statement

In South Africa, the high prevalence of HIV and AIDS has resulted in a significant number of AIDS orphans, which has led to older relatives, particularly grandmothers, taking on extra caregiving roles and assuming the primary responsibility for financial support for their AIDS orphaned grandchildren (Oduaran & Oduaran 2018; WHO 2016). However, government policies and programmes have largely ignored the demands of grandparents caring for AIDS orphans, with the focus being primarily directed towards young people of reproductive age (Kalomo & Liao 2018; Mojola et al. 2021).

As a result, grandparents lack the necessary resources and support to cope with the burden of care as primary caregivers of AIDS orphans, and are often ignored in awareness programmes and support services. Insufficient empirical data exist to guide the development of a comprehensive and sustainable approach that fully supports grandparents in their role as caregivers for AIDS orphans (Mtshali 2016). Hence, this study seeks to thoroughly explore grandparents’ experiences caring for AIDS orphans to acquire baseline data that can contribute in the sustainable development of relevant programmes.

### Study purpose

The study aimed to explore the challenges of grandparents caring for AIDS orphans in the Western Cape province of South Africa.

## Research methods and design

### Study design

The researchers utilised a qualitative approach with an exploratory and descriptive design to explore and depict the challenges faced by grandparents who provide care for AIDS orphans in the Western Cape province, South Africa. The dimensions model of community health nursing (Clark 2003) was employed with a focus on the biophysical, sociocultural, and physical dimensions.

### Setting

The research took place in two districts within the Western Cape province of South Africa: the City of Cape Town Metropolitan and Overberg Municipality, representing both urban and rural areas. Three specific sub-districts were chosen for the study: Khayelitsha and Mitchell’s Plain, located in the urban region of Cape Town, and Grabouw in the rural farming area of the Overberg Municipality. These sites were selected based on factors such as the high prevalence of HIV infection, elevated poverty rates, low educational attainment, and significant crime rates (Simbayi et al. 2019), which contribute to a significant number of orphans within these communities.

### Population and sampling

The target population consisted of grandparents aged 50 years and above, who were caring for AIDS orphans affiliated with NGOs and living in the study setting. A purposive sampling technique to select grandparents was followed, and a sample of 25 was achieved based on data saturation. The study included only 25 female grandparents; male grandparents were invited to participate but none were available.

### Data collection and tools

The individual interviews were conducted employing a semi-structured approach. The researchers used an interview guide that consisted of open-ended questions and probing techniques. The interview sessions took place at a convenient location for both the participants and the researchers. The interviews were recorded using a digital audio recorder, with permission obtained from the participants. Each interview lasted between 30 min and 60 min, and the data collection process extended over approximately 12 weeks.

### Data analysis

The data collected from the interviews were transcribed verbatim and analysed using a thematic approach (Creswell & Creswell 2018). Firstly, the data were coded manually, which included field notes, interviews, and reflection notes, into generative categories. The researchers used the ATLAS.ti programme to organise and categorise the information, as well as identify relationships and themes across all the transcripts. Secondly, an independent qualitative research practitioner also analysed the raw data, and a consensus was reached on the generated themes between all parties.

### Trustworthiness

The researchers ensured credibility through member checking and peer debriefing, and maintained active and prolonged engagement with participants. The researcher maintained objectivity during interviews, handled emotions carefully, and an independent coder validated the findings. Transferability was enhanced by using purposive sampling of participants with accurate and available information. Dependability was maintained through the use of an external audit and thorough documentation of the method used. Confirmability was also enhanced through the use of memos, field notes, and an audit trail that included transcriptions, lists of codes, and data analysis.

### Ethical considerations

The Ethics Committee at the University of the Western Cape (HS 16/5/31) approved the study. The social service research ethic committee can be removed. The participants provided their consent by signing the Consent Forms, and data management systems were applied to ensure their anonymity and confidentiality.

## Results

The study involved 25 grandparents, aged 50 to 70 years, with most of them being retired (88%). Of these, four were married, five were divorced, seven were single, and nine were widows. Two grandparents were still employed, one professionally and the other in unskilled labour. They had cared for orphaned grandchildren for aged 2 years to 20 years. Most grandparents cared for one to four orphaned grandchildren, with 70% testing HIV-negative and 30% testing HIV-positive. Through the analysis of the data, three themes were identified including general health responses, lack of support structures, and challenges related to the behaviour of the orphans.

### Theme 1: General health response

The study participants discussed the increased health risks associated with caring for AIDS orphans as elderly caregivers. They described the challenging and detrimental nature of caregiving tasks, which have negative impact on their general health. Many grandparents even reported being diagnosed with heart disease and acknowledged the immense stress of caregiving on their general health.

#### Subtheme 1.1: Biophysical health

The study discovered that grandparents who care for orphaned grandchildren, specifically those living with AIDS, experience exhaustion because of carrying out household chores alone and a decline in physical strength, which often results in medical conditions. Caring for children living with HIV entails handling additional responsibilities such as going to regular medical appointments, which further intensify their challenges. Some participants shared their experiences, highlighting the difficulties of performing activities such as bathing, feeding, toileting, potty training, and transportation to school and clinics. Two of the participants were recorded saying:

‘My body gets tired. I went to the clinic and they explained to me that I am starting to suffer from heart disease, my legs started to be weak and now I am having the flu, honestly, I don’t feel well.’ (P3; Age 75; Female)

‘I feel tired from looking after them [*AIDS orphaned grandchildren*]. I have to wash their clothes and cook for them, the young one needs to be washed as well and my back gets very painful. Also, my knees are starting to pain.’ (P12; Age 60; Female)

#### Subtheme 1.2: Emotional health

In this study, participants shared that the death of their children had a significant impact on their psychological well-being, leading to a decline in their health. Many participants struggled with sleeping because of constant anxiety regarding the future of their orphaned grandchildren, especially if the grandparents were to pass away. The participants expressed feelings of powerlessness as they were still grieving for their children while also caring for their critically ill grandchildren. One participant reported feeling depressed and abandoned, questioning their circumstances:

‘I think too much and now I am getting sick when I see my child losing her young life in front of me and I cannot help her. She is suffering and her body is finished. I feel helpless and feel bad. I always ask myself why me? Why only my children would die of AIDS. I think I am cursed to get old without children.’ (P23; Age 65; Female)

#### Subtheme 1.3: Social isolation

The act of disclosing their orphaned grandchildren’s HIV status impacted the social interactions of participants with family members and friends, as per their accounts. They stated feeling socially isolated and that their parental responsibilities affected the time they could spend with their friends who did not share similar responsibilities:

‘I feel very alone in this world. My daughter has passed away, and my family is too busy to visit. I spend most of my days inside with the children, and it’s hard to find time to leave the house and socialise. I wish I had more support in my life.’ (P10; Age 69; Female)

The stigma surrounding HIV and/or AIDS resulted in their social isolation from the community, neighbours, and even family members. One participant expressed concerns about potential mockery from others when disclosing their grandchildren’s HIV and/or AIDS status and the cause of their children’s death. A participant expressed this sentiment by stating:

‘No one knows [*the HIV status of the orphaned child*], only the family members. They would mock her [*the HIV-positive grandchild*] if they could know from the area. Even when her eyes were red, they used to mock her at school. Then, I told her that if they mock you again, just tell them you are loved at home.’ (P4; Age 67; Female)

### Theme 2: A lack of support structures

The study participants reported struggling to access both formal and informal support while providing full-time care for their orphaned grandchildren. This had a significant impact on their financial situation. Although they received financial assistance from social services in the form of child support and pension grants, it proved insufficient to meet their monthly expenses, including food, electricity, education, and healthcare for both themselves and the orphans under their care. Their advanced age and limited income made it difficult to meet basic needs, resulting in financial hardship for both grandparents and grandchildren.

#### Subtheme 2.1: A lack of support from family, friends and community

The participants shared statements expressing a deep sense of isolation and helplessness, as they felt abandoned by their own families and burdened with the responsibility of caring for children in their old age. One of the shared statement is as follows:

‘To tell you the truth, this was a big shock in my life. I was not expecting to get old like this, but what shocked me most is the way my daughter is behaving. She doesn’t want to help me to raise her own sister’s kids, even when she was still in the hospital, I had to look after her myself. Nobody wanted to help her and touch her things because she had TB, but nothing happened to me, my child died a very painful death.’ (P20; Age 71; Female)

#### Subtheme 2.2: Difficulty in accessing support from the government

Participants stated that they heavily relied on social support grants as their primary financial source. However, many encountered difficulties while trying to access these grants because of the complex and time-consuming application procedures. Grandparents reported being sent from one office to another, causing further frustration and delays in receiving the support they desperately needed to raise their orphaned grandchildren:

‘I applied for a child support grant for my sick grandchild, and I gave the documents two years ago, but I am still waiting for the money. They would send me up and down telling me to wait. The doctor from side C clinic wrote a letter, and even from that letter still I am not getting the money.’ (P25; Age 60; Female)

The participants disclosed that the lack of essential documentation such as birth certificates and ID hindered their ability to apply for social grants, creating a significant barrier to accessing financial support. A considerable number of the grandchildren were born to unmarried mothers who chose not to reveal the identities of the biological fathers, posing challenges for the grandparents in obtaining the necessary documentation. With the passing of the single mothers, the grandparents were left to care for the orphans without the resources to fulfil their responsibilities:

‘The only thing I always talk about is one thing; the foster care grant process application is delayed because I cannot get documents, and it is not fair that I have to go through all this hardship without help at my age.’ (P21; Age 67; Female)

‘To be honest with you, I don’t even know who the father of my grandchildren is. My daughter never told me who was the father [*before the death of the daughter*], she had different boyfriends and I don’t know who to blame. As for the girlfriend of my son, she is not responsible, she came and dropped the baby off with my son and since then she never turned back. I heard that she is always drunk and use the drug. My grandchildren are better off with me. They’ve suffered enough.’ (P22; Age 72; Female)

#### Subtheme 2.3: Inconsistent support from the non-governmental organisations

Participants in the study reported that NGOs played a significant role in providing support to them, including the provision of food parcels which helped improve the adherence of children living with AIDS to their medication. Participants also reported receiving emotional support through support groups specifically created by NGOs for grandparents caring for orphaned children with AIDS. Additionally, NGOs assisted with the process of applying for social grants and followed up on applications. However, participants also reported that the support received from NGOs was inconsistent and discontinuous, leaving them puzzled about the reasons for the short-term and limited nature of the assistance provided:

‘I received it [*foster care grant*] because of the support group at Umthawelanga. It wasn’t easy to get it [*foster care grant*], but Umthawelanga tried to support me. I didn’t even know that the grandchildren were supposed to get foster care because my own children never got the grant. So, I received this grant because of the sick child.’ (P4; Age 67; Female)

‘They used to call us at the end of the year to give us food and clothes, and now I don’t know why they stopped …’ (P21; Age 67; Female)

### Theme 3: Behavioural challenges with teenage orphans

The majority of grandparents who were taking care of teenage orphans reported experiencing frustration and hopelessness in managing their behaviours and enforcing discipline. The grandparents expressed that behavioural issues such as disobedience and lack of interest in schooling were particularly challenging, causing them significant stress and making it difficult for them to cope.

#### Subtheme 3.1: Disobedience, alcohol and drug use amongst teenagers raised by grandparents

The participants noticed that orphaned teenagers often struggle with alcohol and drug addiction, which adversely affects their academic performance. Some grandparents shared that excessive drinking and smoking on school premises led to their grandchildren’s expulsion. Following are the quotes from two participants describing their experiences:

‘I am having trouble with the teenager, he started smoking, and he is not listening to me, at school, I was called because he was caught dagga smoking at school, and he was given the last warning, but I am very worried about his future, he is not interested in school.’ (P20; Age 71; Female)

‘The last two grandchildren are doing nothing. They don’t want to go to school, they are just sitting at home. At times at school, they would call me complaining about the behaviour of these children because they are on drugs.’ (P14; Age 69; Female)

#### Subtheme 3.2: Increasing demands from teenagers

The participants expressed their worries about the high expectations and demands they faced from the teenagers they were taking care of. The teenagers would request costly items from their grandparents, which they could not afford because of their low income. As a result, some of the teenagers would exhibit troublesome and defiant behaviours, such as refusing to do household chores, engaging in substance abuse, indulging in risky sexual behaviour, and spending excessive amounts of time on their phones. One participant shared her personal experience in this regard:

‘These children want everything, and the government says we must save money for them so we cannot use all of the money [*money from child support grant*], but the money is not even enough. I am not earning a pension grant. My son gets temporally jobs, so he supports us at that particular time when he got lucky. The same child who wants to go to a farewell is asking for a cell phone. I asked if she wants them both and she said “yes”. Then, I told her she needs to choose one. She said, “That means am going to have a cell phone in a long time.” I think she was asking me all this because she knows that they are getting money, yet they don’t even know how small the money is …’ (P6; Age 56; Female)

#### Subtheme 3.3: Truancy as a precursor to poor academic performance of orphans

Some participants reported that their orphaned grandchildren exhibited unacceptable behaviours that resulted in their poor academic performance. One participant shared an experience where she was unaware that her grandchildren were not attending school, which led to their failure:

‘The two children did not do well [*in school*]. I have their reports with me. I went to school and talked with their teachers, so we are helping each other with their schoolwork, I was told from school that they tend to be absent a lot from school [*both grandchildren*]. I did not know that because I send them to school. When they are going to school, they were lying they would go to their friends and watch movies [*instead of attending school*]. The teacher also told me not to give them money and I told the kids that they are going to get nothing from me because when I give them money they don’t come early from school.’ (P24; Age 74; Female)

## Discussion

The study explored and described the challenges that grandparents encounter while taking care of AIDS orphans, particularly those living with AIDS. The caregiving responsibilities and obligations placed on grandparents are arduous, and they must carry them out under exceedingly resource-constrained conditions that undermine their well-being in all aspects (physical, emotional, and socioeconomic).

The main challenge identified in this research is that grandparents are facing extreme poverty, which puts immense pressure on them. Insufficient income prevents them from fulfilling the necessities of the orphans under their care, leading them to heavily rely on social grants for their survival. These findings align with a previous study conducted by Damian et al. (2019) in the Vhembe District of South Africa, which revealed that grandparents caring for AIDS orphans are financially incapable of meeting their grandchildren’s basic and healthcare needs. Similar conclusions were drawn in studies conducted by Carter and Van Breda (2016), Fortune (2016), Böning and Ferreira (2013), Blackie (2014), and Van Der Walt (2018).

The study additionally exposed that the main contributing factor to the poverty is the lack of employment from the grandparents’ side and the lack of financial resources left behind by the deceased parents of the orphaned children. It was shown in the study that the majority of these parents had no means to provide for their children before their death. This finding aligns with the research conducted by Manthosi (2020), which exposed that a majority of the parents of teenage orphaned foster children were unemployed and did not leave any inheritance for their children. Thus, these circumstances result in economic shocks and an increase in poverty levels within the households headed by grandparents. Furthermore, the situation is compounded by the grandparents’ unemployment and their advanced age, which limits their ability to generate income.

Furthermore, the lack of formal and informal support systems is another challenge the grandparents are facing. The study reveals that the majority of family members and the community are not willing to assist grandparents in their caring responsibilities. Also, the study acknowledges the support provided by some NGOs although it is considered occasional, unreliable, and insufficient. These results concur with the results of previous research performed by Matovu and Wallhagen (2020), which emphasised how in their study, grandparents reported receiving irregular, inadequate and inconsistent support from their social network, including adult children, grandchildren, spouses, extended family members, friends, neighbours, and community-based organisations. It was also observed that the support was in many forms, such as loans, food support, monetary assistance, and emotional forms of support.

Receiving social grants poses an enormous challenge for grandparents, mostly because of the struggle of providing the necessary documentation required to qualify for such social grants (such as ID and Birth certificates). It was observed in the study that this problem arose from the lack of disclosure of the identities of the biological fathers while the parents were still alive, resulting in incomplete or rejected social grant applications. A comparable study conducted by Mashegoane and Mohale (2016) in Lephalale, South Africa, found that 40% of participants were unaware of the fathers of their grandchildren, and 30% reported cases where alleged fathers denied paternity despite strong indications suggesting their potential fatherhood. However, in contrast, a study by Kasedde et al. (2014) in Uganda exposed that some parents took deliberate actions to plan and secure the well-being of their children in the event of their passing. In their study, it was observed that before their death, the parents managed to communicate their plan to the grandparents, whom they entrusted with the crucial responsibility of taking care of their children and meeting their needs after their death. This process established a strong support system for the grandparents dedicated to protecting and providing for AIDS orphans.

Another barrier to accessing social grants was the absence of effective service provision during the application process for social grants, resulting from inadequate communication between social service staff and grandparents. This issue further compounds the challenges experienced by grandparents, as they find themselves caught in a cycle of unclear communication with social development officials and they find themselves moving back and forth. The root of this problem is connected to the fact that grandparents are unaware of the specific types of grants they can apply for. These findings align with a similar study conducted by Phetlhu and Watson (2014) in the Koster area of the North West province, South Africa, where grandparents encountered hurdles in accessing social grants because of unsatisfactory service delivery from social development services.

This study revealed that grandparents experienced negative biophysical effects, and these findings are the results of the demanding and strenuous nature of providing constant care to the children, which is physically exhausting. It was observed in the study that many of these grandparents experience adverse consequences on their physical health and immune systems and grandparents often neglect their well-being in the process. The study reported that the majority of these grandparents suffer from bodily pains, uncontrolled high blood pressure, and diabetes mellitus. These findings are consistent with previous research that has established a link between ongoing caregiving and various health issues, including backaches, cardiovascular disease, emotional stress, burnout, fatigue, and bodily pain. An additional study conducted by Osafo et al. (2017) in Uganda highlighted that the demanding responsibilities of caring for orphans may lead to delays in seeking self-care and a low priority placed on the health of grandparents which concurs with the results of this study.

Emotional decline and social isolation were also identified among the challenges grandparents encounter mostly because of the stigma associated with HIV and/or AIDS. They reported feelings of anxiety and fear, starting from concerns about potential discrimination from others in their community, impacting negatively on their emotional well-being. Furthermore, they experienced ongoing grief for their deceased children and were constantly worried and anxious about the future of their orphaned grandchildren and what would happen to them once the grandparents were no longer able to care for them. Similar findings were observed in studies conducted by Mashegoane and Mohale (2016) in South Africa, where mental health decline and emotional distress were reported as the primary challenges faced by grandparents caring for AIDS orphans.

The emotional decline experienced by grandparents is further exacerbated when they face challenges in handling difficult and rebellious teenagers. Because of their advanced age and a lack of energy and skills, grandparents struggle to effectively discipline teenagers, leading to increased stress. The behavioural issues exhibited by teenagers involve engaging in misconduct within the school setting, which can result in academic difficulties, grade repetition, and even school dropout. These findings are consistent with similar studies conducted by Manthosi (2020).

## Conclusion

In summary, the study highlights the significant impact of caring for AIDS orphans on grandparents, who face numerous challenges in fulfilling this role. Financial difficulties, stigma, and a lack of family support contribute to their emotional and physical decline. The demanding caregiving responsibilities limit their engagement in various activities and restrict their leisure pursuits. This adds to the burden they face as they struggle to meet the basic needs of the children. While non-profit organisations provide some assistance, government support is perceived as inadequate. The absence of sufficient policies and programmes further exacerbates the situation. The study recommends the establishment of comprehensive support systems to address the overall well-being of grandparents caring for AIDS orphans in the Western Cape province.
